# Clozapine Long-Term Treatment Might Reduce Epigenetic Age Through Hypomethylation of Longevity Regulatory Pathways Genes

**DOI:** 10.3389/fpsyt.2022.870656

**Published:** 2022-05-18

**Authors:** Blanca Estela Pérez-Aldana, José Jaime Martínez-Magaña, Yerye Gibrán Mayén-Lobo, David José Dávila-Ortiz de Montellano, Carlos Luis Aviña-Cervantes, Alberto Ortega-Vázquez, Alma Delia Genis-Mendoza, Emmanuel Sarmiento, Ernesto Soto-Reyes, Isela Esther Juárez-Rojop, Carlos Alfonso Tovilla-Zarate, Thelma Beatriz González-Castro, Humberto Nicolini, Marisol López-López, Nancy Monroy-Jaramillo

**Affiliations:** ^1^Doctorado en Ciencias Biológicas y de la Salud, Universidad Autónoma Metropolitana, Mexico City, Mexico; ^2^Laboratorio de Genómica de Enfermedades Psiquiátricas y Neurodegenerativas, Instituto Nacional de Medicina Genómica, Mexico City, Mexico; ^3^Departamento de Sistemas Biológicos, Universidad Autónoma Metropolitana, Mexico City, Mexico; ^4^Departamento de Genética, Instituto Nacional de Neurología y Neurocirugía Manuel Velasco Suárez, Mexico City, Mexico; ^5^Departamento de Psiquiatría, Instituto Nacional de Neurología y Neurocirugía Manuel Velasco Suárez, Mexico City, Mexico; ^6^Dirección General, Hospital Psiquiátrico Infantil Juan N Navarro, Mexico City, Mexico; ^7^Departamento de Ciencias Naturales, Universidad Autónoma Metropolitana, Unidad Cuajimalpa, Mexico City, Mexico; ^8^División Académica de Ciencias de la Salud, Universidad Juárez Autónoma de Tabasco, Villahermosa, Mexico; ^9^División Académica Multidisciplinaria de Comalcalco, Universidad Juárez Autónoma de Tabasco, Comalcalco, Mexico; ^10^División Académica Multidisciplinaria de Jalpa de Méndez, Universidad Juárez Autónoma de Tabasco, Jalpa de Méndez, Mexico; ^11^Grupo de Estudios Médicos y Familiares Carracci, Mexico City, Mexico

**Keywords:** DNA methylome, clozapine, psychotic disorders, epigenetic age, longevity

## Abstract

Long-term studies have shown significantly lower mortality rates in patients with continuous clozapine (CLZ) treatment than other antipsychotics. We aimed to evaluate epigenetic age and DNA methylome differences between CLZ-treated patients and those without psychopharmacological treatment. The DNA methylome was analyzed using the Infinium MethylationEPIC BeadChip in 31 CLZ-treated patients with psychotic disorders and 56 patients with psychiatric disorders naive to psychopharmacological treatment. Delta age (Δage) was calculated as the difference between predicted epigenetic age and chronological age. CLZ-treated patients were stratified by sex, age, and years of treatment. Differential methylation sites between both groups were determined using linear regression models. The Δage in CLZ-treated patients was on average lower compared with drug-naive patients for the three clocks analyzed; however, after data-stratification, this difference remained only in male patients. Additional differences were observed in Hannum and Horvath clocks when comparing chronological age and years of CLZ treatment. We identified 44,716 differentially methylated sites, of which 87.7% were hypomethylated in CLZ-treated patients, and enriched in the longevity pathway genes. Moreover, by protein–protein interaction, AMPK and insulin signaling pathways were found enriched. CLZ could promote a lower Δage in individuals with long-term treatment and modify the DNA methylome of the longevity-regulating pathways genes.

## Introduction

There is evidence of premature mortality and an increase in physical comorbidity in patients with psychiatric disorders (1). On average, their life expectancy is reduced from 12 to 15 years compared with the general population (2, 3). Some authors have proposed that the increased mortality could correlate with increased biological aging (4–6). Various epigenetic age estimators of biological age have been developed based on DNA methylation status across a set of CpG sites in cells/tissues, and these have been shown to correlate with the risk of developing age-related conditions and diseases (7–9). Moreover, the presence of psychotic symptoms is observed in different psychiatric illnesses (mainly schizophrenia and bipolar disorder), contributing to this mortality. A recent study reported a positive correlation between epigenetic aging and psychosis severity (10). Fortunately, psychotic symptoms can be controlled with the use of antipsychotics, which also help in reducing the risk of psychotic relapse (11).

On the other hand, the adverse cardiac and metabolic effects of antipsychotics are well known. However, their use in patients with schizophrenia is associated with lower mortality (12). There is evidence demonstrating a 30–50% lower risk of mortality related to the use of antipsychotics compared with non-use (13, 14). In particular, the long-term use of clozapine (CLZ) has been associated with the lowest mortality (12, 15, 16). CLZ is the antipsychotic of choice for patients with refractory psychosis (17), for reducing suicide, and for improving longevity (18). Epidemiological data (19, 20) have supported this last point. Some studies suggest that CLZ promotes epigenetic changes with pro-longevity effects (21, 22), and a recent work has proposed that CLZ may reduce epigenetic aging (23).

The present work aimed to evaluate epigenetic age and DNA methylome differences between patients treated with CLZ and psychiatric patients without psychopharmacological treatment.

## Materials and Methods

### Sample Population

In this study, 87 individuals of Mexican ascendancy were included. Of them, 31 were diagnosed with refractory psychosis and treated with CLZ (CLZ-treated patients), and 56 had early onset psychiatric disorders. This last group of patients has not had any previous psychopharmacological treatment and will be called as drug-naive patients. Samples from both groups of patients were recruited from the outpatient clinics. The CLZ-treated patients with refractory psychosis were recruited from the Instituto Nacional de Neurología y Neurocirugía Manuel Velasco Suárez (INNNMVS), and drug-naive patients from the Hospital Psiquiátrico Infantil Juan N. Navarro and the Hospital General Dr. Gustavo Rivorosa Pérez. At least one specialized psychiatrist performed the diagnosis. Individuals with intellectual disabilities, heavy drinkers, smokers, or substance abusers were excluded. In addition, we excluded drug-naive patients who had begun psychopharmacological treatment. The study was conducted according to the guidelines of the Declaration of Helsinki and approved by the Institutional Research and Bioethics Committees of INNNMVS (protocol 38/19), the Ethics Committee of the Children’s Psychiatric Hospital Dr. Juan N. Navarro with approval No. II3/01/0913 (11 October 2017), and by the Ethics Committee of the National Institute of Genomic Medicine (INMEGEN) with approval No. 06/2018/I. Furthermore, all individuals signed informed consents. For those patients under the age of 18 years, an informed assent letter was signed by both parents/guardians and the minor.

### Analysis and Quality Control of Microarrays

Blood DNA extraction, bisulfite-conversion (Zymo, Irvine, CA, United States), and microarray hybridization (Infinium MethylationEPIC BeadChip, Illumina, San Diego, CA, United States) were performed according to the manufacturer’s protocol. We used the Genome Reference Consortium Human Build 37 (GRCh37/hg19) as position reference for all the analyses. The fluorescence intensities were measured with the iScan instrument and transformed into idat files with the algorithm implemented in the GenomeStudio software (Illumina, San Diego, CA, United States). Specialized staff carried out microarray analyses in the INMEGEN. The ChAMP (24) package was used for quality control. Briefly, the probes using the following filters were removed: (i) detection *p* greater than 0.01, (ii) less than three beads in less than 5% of the samples, (iii) all non-CpG sites, (iv) single nucleotide polymorphism (SNP)-associated probes, (v) probes associated with sex chromosomes, and (vi) multi-hit probes. In addition, samples with a ratio greater than 0.1 were removed, resulting in one individual filtered out. After quality control, a matrix of beta values was constructed.

### Epigenetic Age Calculation

We normalized the previous beta value matrix by beta-mixture quantile normalization (25) and calculated epigenetic ages with the *ENmix* package (26). This package allowed us to calculate three epigenetic clocks: Hannum (7), Horvath (8), and DNAm PhenoAge (9). The epigenetic ages calculated with the Horvath, Hannum, and PhenoAge clocks and the chronological age in both groups were compared by correlation analysis. Then, we calculated the delta of age (Δage = epigenetic age − chronological age), subtracting the chronological age from the estimated epigenetic age in all samples and by each clock. Finally, we compared the Δage of each clock between CLZ-treated patients and drug-naive patients. First, stratified analysis by sex and age was carried out, comparing the CLZ-treated patients vs. drug-naive patients. The CLZ-treated patients’ group was later subdivided into subgroups according to the time’s mean of treatment with CLZ (>9 and ≤ 9 years of treatment; i.e., few and many years of treatment, respectively) and according to the mean chronological age (cut-off value = 37 years; i.e., young and older patients). Due to the small sample size, and to achieve subgroups with a similar number of patients (*n*), the mean values of the years of CLZ treatment and chronological age of patients were used. The student’s *t*-test was applied for the analysis of these four subgroups.

### Differentially Methylation Sites Analysis

We removed the batch effect and adjusted blood cell proportion on the normalized beta values matrix with a single-value deconvolution (*champ.SVD* and *champ.runCombat*) and a reference base analysis (*champ.refbase*), respectively (24, 27). Once adjusted, we analyzed differentially methylated sites analysis by linear models implemented in *champ*.DMP function of the *ChAMP* package (28). First, we analyzed differentially methylated sites considering significance at a *p* lower than 1e-08 (a summary of statistics is available in Supplementary Table 1). Then, after identifying differentially methylated sites, a differentially methylated region analysis was carried out with the ProbeLasso algorithm (29) (a summary statistics of the differential methylation region analysis is available in Supplementary Table 2).

### Pathway Enrichment and Protein-Protein Interactions

We performed pathway enrichment after extracting genes resulting from differentially methylated sites and regions through the WebGestalt platform (WEB-based GEne SeT AnaLysis Toolkit) with free access (30) (all pathways of this enrichment are available on Supplementary Table 3). First, we analyzed protein–protein interactions between gene products identified using the web application String (31) and plotted the interactions with Cytoscape 3.8.1 software (32). Then, a sub-enrichment analysis of the longevity regulating pathway was performed in String using the KEGG pathway database (33).

## Results

### Study Population

In this study, eighty-seven individuals were included and classified into two groups: the CLZ-treated patients with refractory psychosis (*n* = 31), and the drug-naive patients with psychiatric disorders (*n* = 56) (Table 1). The following comorbidities were observed in CLZ-treated patients: diabetes (22.6%), epilepsy (16.1%), hyperlipidemia (12.9%), hypercholesterolemia (6.4%), hypothyroidism (6.4%), hypertension (9.7%), and obsessive-compulsive disorder (3.2%). Details about the comorbidities found in the naive patients have been previously reported (34).

**TABLE 1:** Clinical and demographic characteristics of all patients.

Characteristic	CLZ-treated patients (*n* = 31)	Drug naive patients (*n* = 56)
Age in years (mean ± DE)	37.74 ± 12.18	17.12 ± 7.84
**Sex (%)**		
Male	18 (58.1)	13 (23.2)
Female	13 (41.9)	43 (76.8)
**Diagnosis (%)**		
Major depressive disorder	0	19 (33.9)
Attention-deficit hyperactivity disorder	0	32 (57.1)
Schizophrenia/early onset psychosis	19 (61.3)	5 (9.0)
Bipolar Disorder	3 (9.6)	0
Schizoaffective Spectrum Disorder	8 (25.8)	0
Psychotic Depressive Episode	1 (3.2)	0
**Comorbidities (see text)**		
Yes	19 (61.3)	50
No	12 (38.7)	6
CLZ Treatment years (mean ± DE)	9.22 ± 7.54	0 (0.0)
**Concomitant treatment (%)**		
Yes	29 (93.6)	0 (0.0)
Anxiolytics	17 (54.8)	0 (0.0)
Antidepressants	20 (64.5)	0 (0.0)
Anticonvulsants^+^	13 (41.9)	0 (0.0)
Anticholinergics	2 (6.4)	0 (0.0)
No	2 (6.4)	56 (100)
**Alcohol consumption (%)**		
Yes	10 (32.3)	0 (0.0)
No	21 (67.7)	56 (100)
**Drugs consumption (%)**		
Yes	8 (25.8)^¥^	0 (0.0)
No	23 (74.2)	56 (100)
**Tobacco use (%)**		
Yes	14 (45.2)	0 (0.0)
No	17 (54, 8)	56 (100)
**CLZ dosage (mg/day)**	207.76 ± 128.71	0 (0.0)

*^¥^These patients were former consumers of marijuana. ^+^Including 1/1/8 patients receiving carbamazepine/phenytoin/valproate. SD, standard deviation.*

### Epigenetic Age in Clozapine-Treated Patients vs. Drug-Naive Patients

**FIGURE 1:**
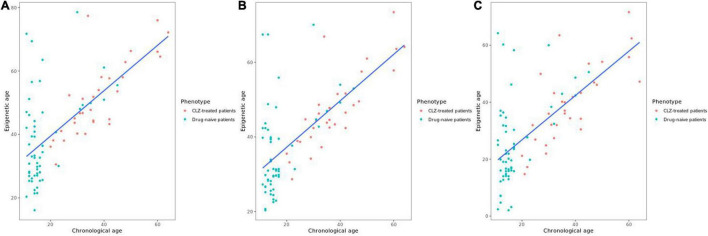
Correlation between chronological age and epigenetic age. **(A)** Correlation with Horvath’s clock. **(B)** Correlation with Hannum’s clock. **(C)** Correlation with PhenoAge’s clock.

**FIGURE 2:**
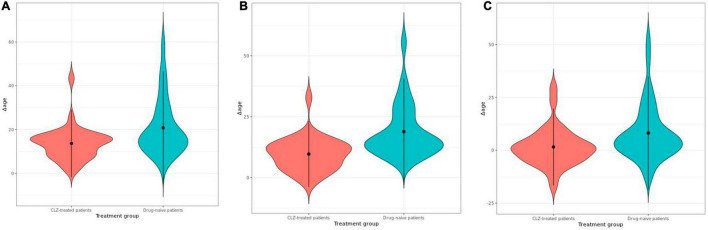
Violin plots showing the comparison of Δage between clozapine (CLZ)-treated patients and drug-naive patients estimated by using epigenetic clocks of **(A)** Horvath, **(B)** Hannum, and **(C)** PhenoAge.

The variable sex did not show a significant correlation with epigenetic age; however, it was considered in the model as previous reports have demonstrated differences in the epigenetic age between men and women (23). The years of CLZ treatment correlated with epigenetic age in the PhenoAge clock (*t* = 2.6041, *r*^2^ = 0.4415, *p* = 0.01458); thus, these two variables were included in the stratification of the patients. The chronological age positively correlated with the epigenetic age in all clocks, Hannum (*r*^2^ = 0.679, *p* = 4.96e-13), Horvath (*r*^2^ = 0.662, *p* = 2.85e-12), and PhenoAge (*r*^2^ = 0.664, *p*-value = 2.26e-12) (Figure 1). After obtaining the Δage for all samples, we found that the CLZ-treated patients’ group had a smaller Δage compared with drug-naive patients (Figure 2). This difference was significant for the three analyzed clocks: Horvath (*t* = −3.23, *p* = 0.0018), Hannum (*t* = −4.83, *p* = 6.0740e-06), and PhenoAge (*t* = −2.72, *p* = 0.0079). Hannum’s clock showed the most significant value. The mean Δage in the CLZ-treated patients was 13.64, 9.65, and 1.54 years for Horvath, Hannum, and PhenoAge clocks, respectively. In drug-naive patients, the mean Δage was 20.77, 18.84, and 8.15 years in the same mentioned order for the three clocks.

The stratified analysis by sex and age, after comparing CLZ-treated patients vs. drug-naive patients, showed significant differences in the three clocks only for CLZ-treated male patients with mean Δage values of 13.15, 10.07, and 1.60 years for the Horvath, Hannum, and PhenoAge clocks, respectively, showing corresponding values of *p* of 0.0376, 0.0073, and 0.0370 (Supplementary Figure 1A). The mean Δage in CLZ-treated female patients was 14.30, 9.06, and 1.47 years for the Horvath, Hannum, and PhenoAge clocks, respectively. However, significant differences were only found in Hannum’s clock (*t* = −3.2253, *p* = 0.0035). Thus, we did not identify differences between age subgroups (Supplementary Figure 1B).

### Comparison of Δage in Older and Younger Patients Treated With Clozapine

**FIGURE 3:**
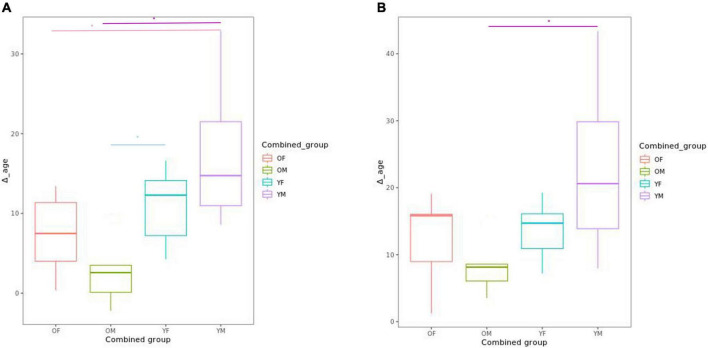
Box plot showing the comparison of delta age (Δage) using **(A)** Hannum’s clock and **(B)** Horvath’s clock in the four groups generated according to the mean values of chronological age and years of CLZ treatment. Differences are observed between young patients with more or a few years of treatment vs. older patients with many years of treatment. The four groups analyzed were: young patients with few years of treatment (YF); young patients with many years of treatment (YM); older patients with few years of treatment (OF), and older patients with many years of treatment (OM). The asterisk (*) denotes significant differences.

We explored Δage differences in CLZ-treated patients, subdivided according to the mean value of chronological age (37.74 years) and the mean treatment time with CLZ (9.22 years). Significant differences of Δage for the Horvath and Hanum clocks were observed between patients by years of CLZ treatment and chronological age (younger vs. older patients, and few years of treatment vs. many years of treatment, respectively). In addition, other significant differences were found for the Hannum clock in the following comparisons: older patients with more years of treatment (>9.22 years) vs. younger patients with more years of treatment (*p* = 0.0021); older patients with many years of treatment vs. young patients with few years of treatment (*p* = 0.0263); and older patients with few years of treatment vs. young patients with many years of treatment (*p* = 0.0236) (Figure 3A). Regarding Horvath’s clock, we only observed differences between the group of older patients with many years of treatment and younger patients with many years of treatment (*p* = 0.019) (Figure 3B).

### Differentially Methylated Sites

We identified 44,716 differentially methylated sites between CLZ-treated patients and drug-naive patients. Of note, 39,204 CpG sites (87.70%) were hypomethylated in the CLZ-treated patients, and the remaining (5,512 CpG sites, 12.30%) were hyper-methylated.

According to the position of the differentially methylated sites in relation to the gene structure, the following distribution was observed: 9,573 (24.42%) hypomethylated sites were located at 200 nucleotides of the transcriptional start site (TSS200), 7,247 (18.49%) sites on TSS1500, 7,126 (18.18%) on gene body, 5,332 (13.6%) on 5′-UTR, 4,626 (11.8%) on first exon, 306 (0.78%) on 3′-UTR, and 22 (0.06%) on exon binding sites. The distribution of the hypermethylated sites was as follows: 3,111 (56.44%) sites were annotated to gene body, 266 (4.83%) on 5′-UTR, 265 (4.81%) on TSS1500, 262 (4.75%) on 3′- UTR, 138 (2.5%) were located on the TSS200, 75 (1.36%) on first exon, and 55 (0.1%) on exon binding sites.

The location of differentially methylated sites concerning CpG islands was as follows: 29,895 (66.86%) sites on CpG islands, 7,860 sites (17.56%) on shores, 5,808 sites (12.99%) on the open sea, and 1,153 sites (2.58%) on shelves. Regarding hypomethylated sites, 29,020 of them (74%) were found on CpG islands, 6,822 (17.4%) on shores, 2,892 sites (7.38%) on the open sea, and 470 (1.2%) sites on shelves. The distribution of the hypermethylated sites was: 2,916 (52.9%) sites located on the open sea, 1,038 (18.83%) sites on shores, 875 (15.87%) sites on CpG islands, and 683 (12.39%) sites on shelves.

### Enrichment of the Genes Located Within the Differentially Methylated Sites

**FIGURE 4:**
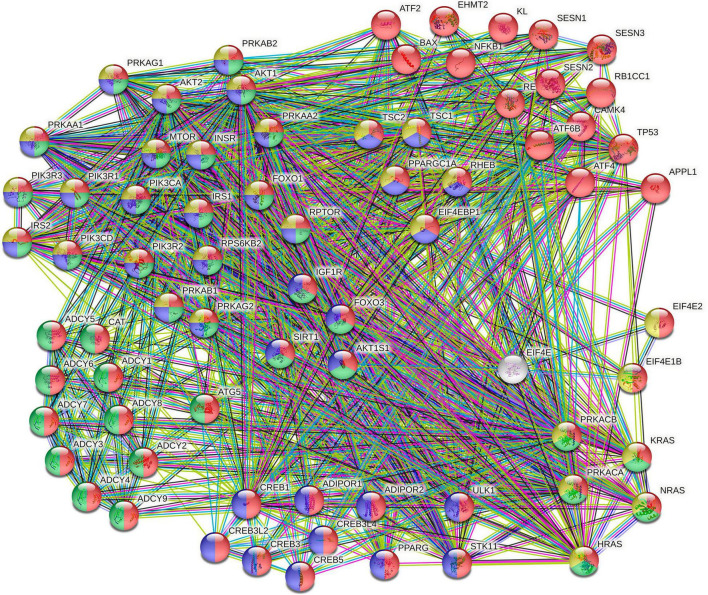
Protein–protein interaction between gene products of the longevity regulating pathway. Clusters are colored based on the Search Tool for Retrieval of Interacting Genes/Proteins (STRING) analysis. The proteins involved in the longevity regulating pathway are highlighted in red and green colors (KEGG pathways: hsa04211 and hsa04213, respectively). In contrast, AMPK signaling proteins (hsa04152) and the insulin signaling pathway proteins (hsa04910) are shown in blue and yellow colors, respectively.

In total, sixty-five pathways enriched at the hypomethylated sites were identified, and the main ones were involved in the biosynthesis of glycosaminoglycan, glioma, longevity regulatory pathway, VEGF signaling pathway, and circadian rhythm. Additionally, the hypermethylated sites are enriched on ABC transporters and endocytosis pathways (Table 2). Supplementary Table 2 contains the enrichment of all the observed pathways. Among the enriched pathways at the hypomethylated sites, the longevity pathway was identified, which, in turn, interacts with others, such as the AMPK and the insulin signaling pathways. The longevity regulating pathway occupied the 21st place of the ranked list generated by the pathway enrichment; it showed an overlap of 74 genes out of an expected 89 [*p* = 1.25e-7, false discovery rate (FDR) = 1.14e-6, and enrichment ratio = 1.4578]. We sub-analyzed the longevity regulating pathway because CLZ has been associated with a reduced mortality risk compared with other antipsychotics. For this purpose, a sub-enrichment analysis using protein–protein interaction on this pathway (Figure 4) was performed.

**TABLE 2:** Summary of the pathway enrichment for hypomethylated and hypermethylated sites.

Gene set	Pathway	Genes*	FDR	Enrichment ratio	No of genes found/expected
**Hypomethylated**					
hsa05213	Endometrial cancer	*AKT1, AKT2, APC, APC2, AXIN1, AXIN2, BAD, BAK1, BAX, BRAF*	2.4e-06	1.542	51/58
hsa03430	Mismatch repair	*EXO1, MLH1, MLH3, MSH2, MSH3, MSH6, PCNA, PMS2, POLD2, POLD3*	6.4e-03	1.525	20/23
hsa05226	Gastric cancer	*ABCB1, AKT1, AKT2, APC, APC2, AXIN1, AXIN2, BAK1, BAX, BCL2*	3.8e-12	1.494	127/149
hsa00532	Glycosaminoglycan biosynthesis	*B3GALT6, B3GAT3, B4GALT7, CHPF, CHPF2, CHST11, CHST12, CHST13, CHST14, CHST3*	1.9e-02	1.490	17/20
hsa05214	Glioma	*AKT1, AKT2, BAK1, BAX, BRAF, CALM1, CALM2, CALM3, CALML4, CALMK2B*	4.4e-06	1.482	60/71
hsa00520	Amino sugar and nucleotide sugar metabolism	*AMDHD2, CMAS, CYB5R1, CYB5R2, CYB5R3, CYB5R4, CYB5RL, FUK, GALE, GALK1*	3.9e-04	1.461	40/48
hsa04211	Longevity regulating pathway	*ADCY1- ADCY9, ADIPOR1, ADIPOR2, AKT1, AKT1S1, AKT2, APPL1, ATF2, ATF4, ATF6B*	1.1e-06	1.458	74/89
hsa04390	Hippo signaling pathway	*ACTB1, ACTG1, AJUBA, APC, APC2, AXIN1, AXIN2, BBC3, BIRC2, BIRC5*	3.2e-10	1.446	127/154
hsa03050	Proteasome	*ADRM1, POMP, PSMA1, PSMA3. PSMA4, PSMA5, PSMA6, PSMA7, PSMB1, PSMB10*	1.1e-03	1.442	37/45
hsa04370	VEGF signaling pathway	*AKT1, AKT2, BAD, CASP9, CDC42, HRAS, HSPB1, JMJD7-PLA2G4B, KDR, KRAS.*	2.8e-04	1.426	48/59
**Hypermethylated**					
hsa02010	ABC transporters	*ABCA1, ABCA12, ABCA2, ABCA3, ABCA7, ABCB8, ABCB9, ABCC1, ABCC12, ABCC2*	1.6e-04	3.402	19/44
hsa04144	Endocytosis	*AGAP1, AP2A1, AP2A2, AP2B1, AP2M1, ARAP1, ARAP3, ARPC1B, ASAP1, ASAP2*	1.6e-04	1.873	244/58

*Only the top 10 genes of the top enriched pathways for differentially methylated sites between CLZ-treated patients and drug-naive patients are shown.

*FDR, False discovery rate; No, number.*

### Differentially Methylated Regions

A total of 286 differentially methylated regions were identified, of which 247 regions were hypomethylated and 39 hypermethylated. Then, the FUMA platform [Functional Mapping and Annotation of Genome-Wide Association Studies (GWAS)] (35) was used to prioritize and interpret GWAS results of the 307 annotated genes in this region. This analysis revealed top genes, such as *NOTCH4, MICA, TRIM27, PBX2*, and *FKBPL*, as enriched in GWAS of schizophrenia, CLZ-induced agranulocytosis/granulocytopenia in treatment-resistant schizophrenia, and bipolar disorder among other psychiatric and non-psychiatric conditions (Supplementary Figure 2). In this enrichment analysis of the differentially methylated regions, we did not find any pathway with a statistically significant association; nevertheless, in the nominal association, twelve pathways were identified (Supplementary Figure 3).

## Discussion

Various analyses of DNA methylome in CLZ-treated patients have been documented in European (36, 37) and Asiatic populations (38, 39). Herein, we report the first differential methylation analysis in a Latin American population between CLZ-treated patients with refractory psychosis and drug-naive patients with psychiatric disorders. Our results showed that the proportion of hypomethylated CpG sites (87.7%) was much higher than hypermethylated ones (12.3%) in the CLZ-treated group. This finding agrees with previous studies that found a higher proportion of hypomethylated sites in patients with refractory schizophrenia treated with CLZ (40), and an increase in the global DNA hypomethylation in leukocytes obtained from patients with schizophrenia who received CLZ (41). Additional studies have reported that CLZ induces DNA demethylation at the level of specific genes; for instance, evidence suggests that CLZ may attenuate the dysregulation of GABAergic and glutamatergic transmission by reducing gene promoter hypermethylation (42, 43). These findings raise CLZ as a potential drug that deserves further investigation and, based on the screening of new potential targets in CLZ, appears to be a promising strategy for drug repurposing.

On the other hand, our results showed that patients with psychosis treated with CLZ had a lower Δage than those without psychopharmacological treatment. In addition, we found a significant reduction in the epigenetic age for the three studied clocks in the group of male patients; in this regard, a previous study observed the same effect of CLZ in reducing epigenetic age in male patients (23). Although the authors studied a larger sample of patients with schizophrenia in that study, they included very few CLZ users and did not perform differential methylation analysis. In this sense, the previous findings are consistent with ours. They suggested that treatment with CLZ could reduce epigenetic aging and directly impact the mortality of individuals treated with this antipsychotic.

Collectively, one of the pathways found to be hypomethylated was the estrogen signaling pathway, including the gene encoding for the estrogen receptor 1 (ESR1) (Figure 3). ESR1 is highly involved in estrogen metabolism, and differences in the frequency of some of its genetic variants have been associated with longevity in humans (44, 45). The DNA methylation levels in this gene may explain the difference in epigenetic age reduction only in men. Estrogen levels in a mouse model treated with 17-alpha estradiol affected metabolic parameters and delayed aging in male mice (46, 47). If CLZ induces DNA hypomethylation, it will increase *ESR1* gene expression and estrogen sensitivity, which could fit in a hypothesis behind the significant epigenetic age reduction observed in our study. The longevity regulating pathway also appeared to be potentially involved in CLZ-induced epigenetic age changes. This is in line with epidemiological studies and a meta-analysis by Vermeulen et al. (48), which have reported that the long-term CLZ treatment is associated with an about 40% lower all-cause mortality compared with other antipsychotics (12, 15, 16, 49). In addition, we were able to identify enrichment of the membrane-bound adenylyl cyclase enzymes (also known as adenylate cyclases) in this pathway. Contradictorily, the hypomethylation of ADCYs genes would have a similar action to that reported when these genes are inhibited. For instance, the *Adcy5* knockout mouse lives longer and is used as a model of longevity (50–54). Taken together, our findings suggest that the regulation of the longevity pathway by CLZ action may promote epigenetic changes that need to be further explored. Interestingly, another group of genes found in the protein–protein interaction network analysis was related to AMP-activated protein kinases. The activation of this pathway has also been associated with longevity and aging delay (55–59).

Clozapine exhibits unique benefits for ameliorating symptoms in patients with treatment-resistant psychosis, for reducing suicide, and for improving longevity (18). This last characteristic has been observed in some epidemiological studies (14, 19, 20, 49), and in a recent study using age classifiers to drug- induced transcriptomes that identified several geroprotectors, such as CLZ (60). Thus, despite lethal adverse reactions of CLZ, this molecule may have pro-longevity effects that should be further investigated.

Some limitations of this study should be considered. First, our sample size is small, however, this is the first study of CLZ effect on DNA methylome in Latin American patients with psychosis, and the number of CLZ-users included in other reports is still restricted and similar in numbers to ours (which is partly due to the misperception that CLZ is a dangerous therapeutic agent). Despite this, some of our results confirm previous findings from other recent studies. Second, our drug-naive patients had different psychiatric disorders and were younger than CLZ-treated patients’ group, which might influence the observed findings. Third, this is a cross-sectional study performed in a peripheral tissue, in which we did not evaluate other variables that may modulate epigenetic aging, such as prior pharmacological interventions or concomitant to CLZ that might impact DNA methylation. For instance, eight CLZ-treated patients were also receiving valproate, a drug that has been reported to be a potent epigenetic agent, affecting the DNA and histone methylation status (61). Nevertheless, due to a paucity of longitudinal epigenetic data of CLZ-treated patients, we believe that this is a good approach to evaluate the potential CLZ effects on the epigenome. In this study, we only evaluated DNA methylome, future longitudinal studies should confirm our findings and explore whether CLZ may be considered as an epigenetic drug. We could conclude that the long-term CLZ treatment might reduce epigenetic aging in male patients with psychotic disorders. In addition, our findings suggest a hypomethylation of genes of the longevity regulatory pathway as a potential action of CLZ. Thus, CLZ poses an exciting mechanism to be further explored to improve life expectancy and ameliorate the aging effects of patients treated with this antipsychotic.

## Data Availability Statement

The data presented in the study are deposited in the GEO online repository, accession numbers GSE197136 and GSE201638.

## Ethics Statement

The studies involving human participants were reviewed and approved by the Research and Bioethics Committees of Instituto Nacional de Neurología y Neurocirugía Manuel Velasco Suárez, the Ethics Committee of the Children’s Psychiatric Hospital Dr. Juan N. Navarro with approval No. II3/01/0913, and the Ethics Committee of the National Institute of Genomic Medicine with approval No. 06/2018/I. Furthermore, all adult participants signed informed consent. For those patients under the age of 18 years, an informed assent letter was signed by both parents/guardians and the minor.

## Author Contributions

NM-J, ML-L, and HN: conceptualization, resources, and project administration. BP-A, DD-O, AO-V, CA-C, YM-L, ES, IJ-R, CT-Z, and TG-C: methodology. BP-A, JM-M, and AG-M: software. BP-A and JM-M: formal analysis and data curation. BP-A, YM-L, AO-V, DD-O, JM-M, AG-M, and ES-R: investigation. BP-A, JM-M, and NM-J: writing—original draft preparation. ES-R, HN, ML-L, and NM-J: writing—review and editing. ES-R, YM-L, AO-V, DD-O, JM-M, AG-M, BP-A, ES, IJ-R, CT-Z, TG-C, NM-J, ML-L, and HN: visualization. NM-J, ML-L, AG-M, and HN: supervision. NM-J, AG-M, ML-L, and HN: funding acquisition. All authors have read and agreed to the published version of the manuscript.

## Conflict of Interest

The authors declare that the research was conducted in the absence of any commercial or financial relationships that could be construed as a potential conflict of interest.

## Publisher’s Note

All claims expressed in this article are solely those of the authors and do not necessarily represent those of their affiliated organizations, or those of the publisher, the editors and the reviewers. Any product that may be evaluated in this article, or claim that may be made by its manufacturer, is not guaranteed or endorsed by the publisher.
