# Supplementary material for: Clozapine Long-Term Treatment Might Reduce Epigenetic Age Through Hypomethylation of Longevity Regulatory Pathways Genes
Source: Front Psychiatry. 2022 May 18;13:870656. doi: 10.3389/fpsyt.2022.870656 (PMC9157596; doi:10.3389/fpsyt.2022.870656)
Supplement: Supplementary file 1 [file Data_Sheet_1.docx]

Supplementary Material

## Supplementary Figures


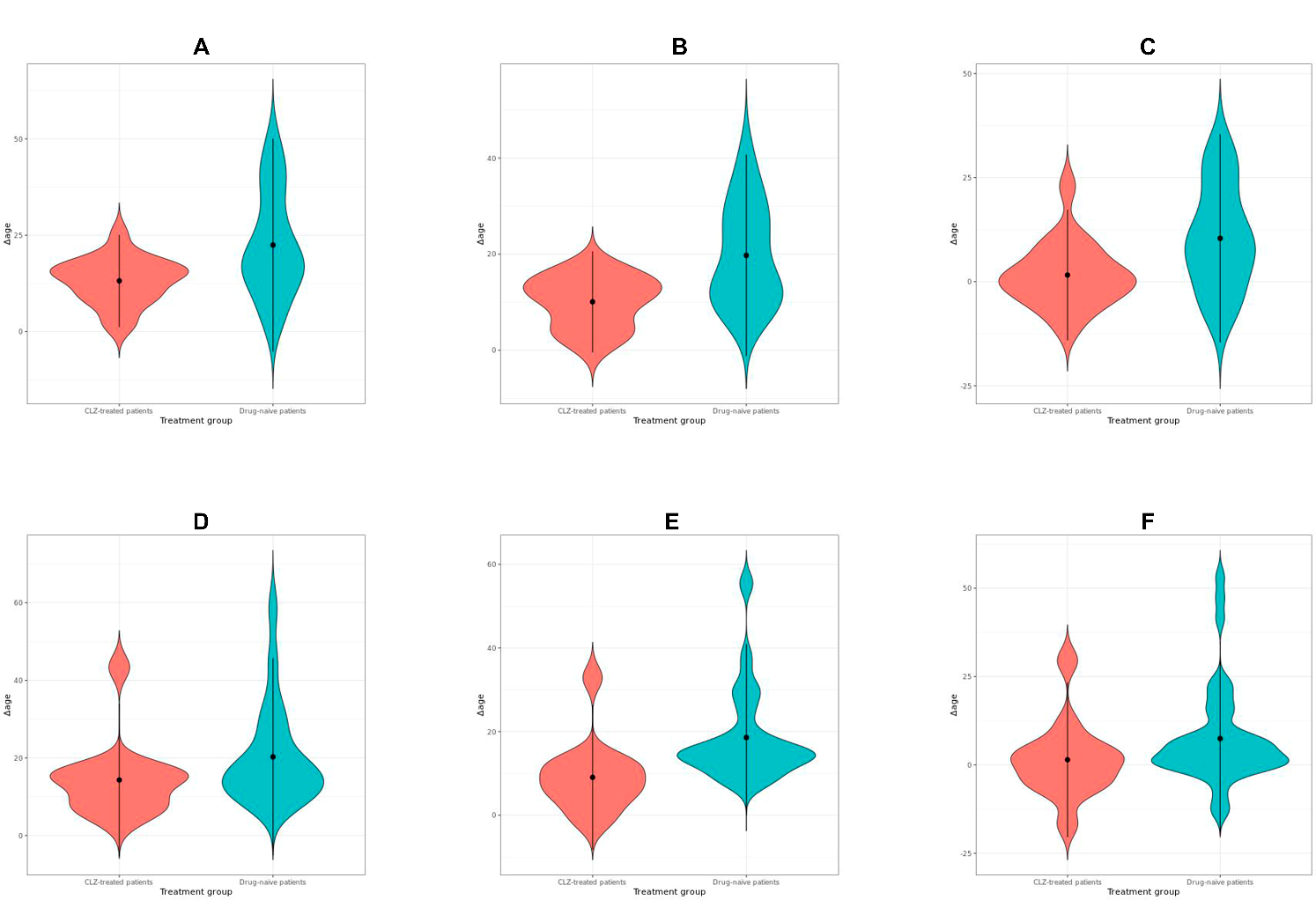


**Supplementary Figure 1.** Violin plots showing the comparison of Δage stratified by gender between CLZ-treated patients and drug-naive patients. **A)** Horvath clock male patients, **B)** Hannum clock male patients, **C)** PhenoAge clock male patients, **D)** Horvath clock female patients, **E)** Hannum clock female patients and **F)** PhenoAge clock female patients.


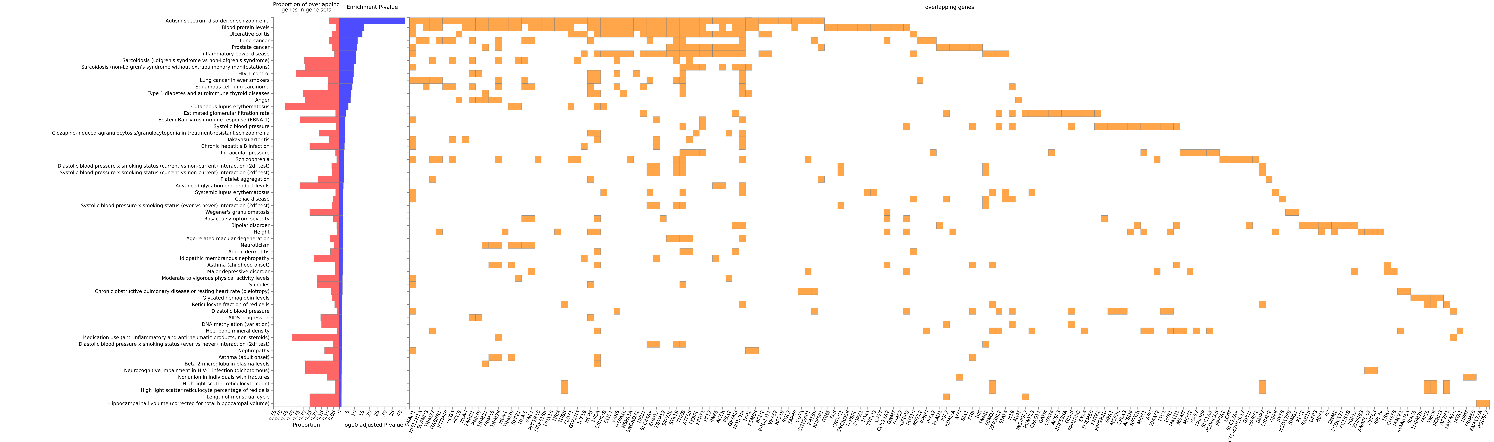


**Supplementary Figure 2.** FUMA analysis performed with the 307 annotated genes found in the differentially methylated regions. Top genes were enriched in GWAS of schizophrenia and autism spectrum disorder. Clozapine-induced agranulocytosis/granulocytopenia in treatment-resistant schizophrenia, and bipolar disorder were also observed.


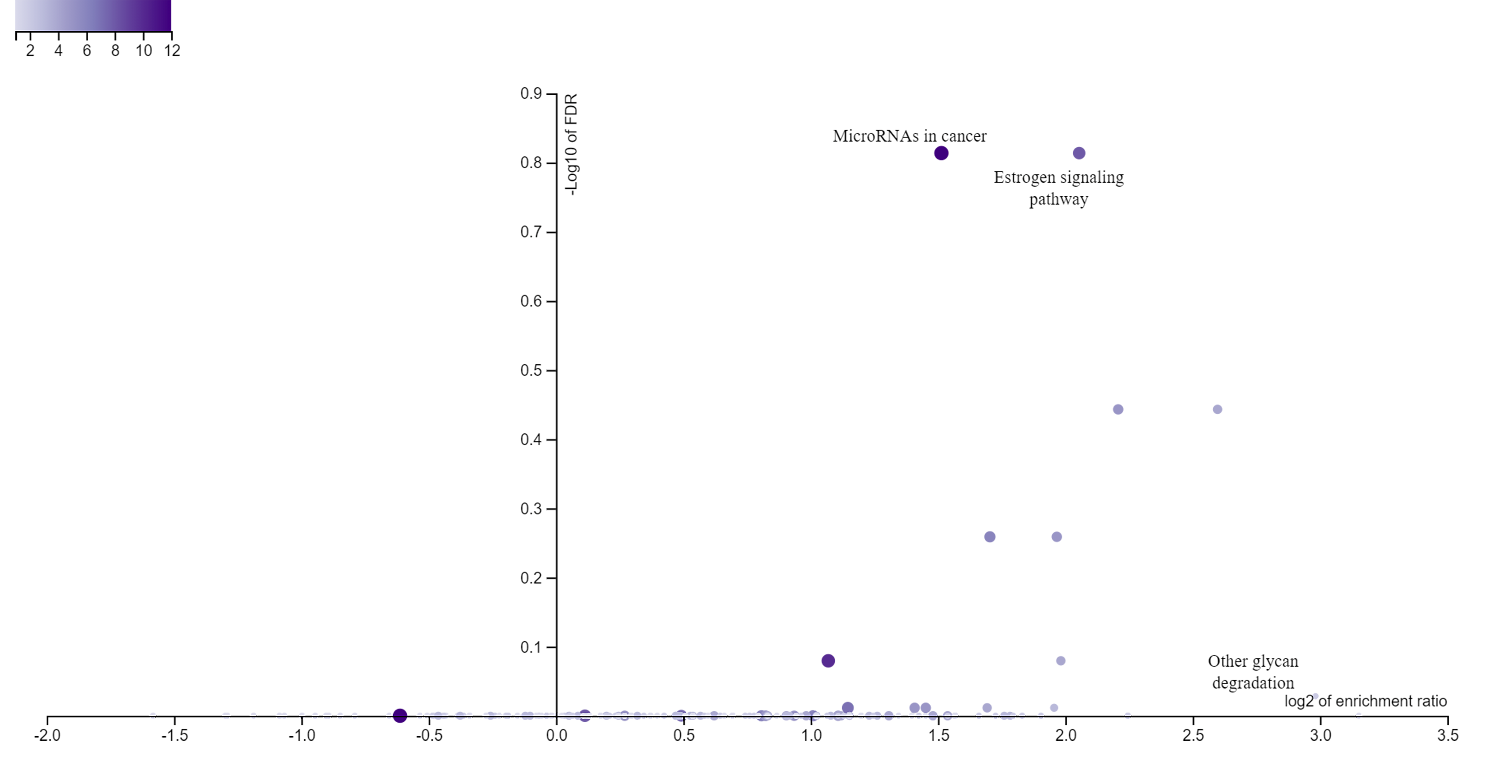


**Supplementary Figure 3.** Volcano plot showing the enriched pathways of the genes located within the differentially methylated regions. On the X-axis, the log2 values of the enrichment ratio vs. the -log10 values of the false discovery rate (FDR) for each pathway are plotted. The size and color of the point are proportional to the size of the category. The upper left scale bar depicts the number of genes obtained from our results for those expected in each pathway. The darker the color, the greater the number of genes identified.

**Supplementary Tables captions**

**Table S1. Summary statistics of the differentially methylated sites**

DMP: Differential Methylation Probes.

ID_CpG: Identifier of the probe specific to a site in the genome.

Clozapine_to_Drug-naive.logFC: Log Fold Change = Log (FC).

Clozapine_to_Drug-naive.AveExpr: Beta values of all individuals.

Clozapine_to_Drug-naive.t: t-value obtained from the comparison between the group of CLZ-treated patients vs Drug-naive patients.

Clozapine_to_Drug-naive.P.Value: p-value obtained from the comparison between the group of CLZ-treated patients vs Drug-naive patients.

Clozapine_to_Drug-naive.adj.P.Val: Adjusted p-value obtained from the comparison between CLZ-treated patients vs Drug-naive patients.

Clozapine_to_Drug-naive.B: log-odds that the site is differentially methylated.

Clozapine_to_Drug-naive.Clozapine_AVG: Beta values of patients under treatment with CLZ.

Clozapine_to_Drug-naive.Drug-naive_AVG: Beta values of drug naive patients.

Clozapine_to_Drug-naive.deltaBeta: Differences in beta values between the two groups.

Clozapine_to_Drug-naive.CHR: Chromosome where the methylation site is located.

Clozapine_to_Drug-naive.Strand: Strand on which the methylation site is located; F= Forward; R=Reverse.

Clozapine_to_Drug-naive.SNP_ID: Single nucleotide variant identifier.

**Table S2. Summary statistics of the differentially methylated regions.**

DMR: Differentially methylated region.

ProbeLassoDMR.dmrP: p-value corresponding to each DMR.

ProbeLassoDMR.dmrChrom: Chromosome on which the DMR is located.

**Table S3. Pathway enrichment of sites with differential methylation.**

geneSet: Identifier of each way.

FDR: False Discovery rate.

userId: Name of genes identified in each pathway.
